# The Development of Biophotovoltaic Systems for Power Generation and Biological Analysis

**DOI:** 10.1002/celc.201900997

**Published:** 2019-09-18

**Authors:** Laura T. Wey, Paolo Bombelli, Xiaolong Chen, Joshua M. Lawrence, Clayton M. Rabideau, Stephen J. L. Rowden, Jenny Z. Zhang, Christopher J. Howe

**Affiliations:** ^1^ Department of Biochemistry University of Cambridge Tennis Court Road Cambridge CB2 1QW UK; ^2^ Department of Chemistry University of Cambridge Lensfield Road Cambridge CB1 2EW UK; ^3^ Dipartimento di Scienze e Politiche Ambientali Università degli Studi di Milano Milan Italy; ^4^ Department of Chemical Engineering and Biotechnology University of Cambridge Philippa Fawcett Dr Cambridge CB3 0AS UK

**Keywords:** biophotovoltaics, biophotoelectrochemistry, electrode architecture, fuel cells, photosynthesis

## Abstract

Biophotovoltaic systems (BPVs) resemble microbial fuel cells, but utilise oxygenic photosynthetic microorganisms associated with an anode to generate an extracellular electrical current, which is stimulated by illumination. Study and exploitation of BPVs have come a long way over the last few decades, having benefited from several generations of electrode development and improvements in wiring schemes. Power densities of up to 0.5 W m^−2^ and the powering of small electrical devices such as a digital clock have been reported. Improvements in standardisation have meant that this biophotoelectrochemical phenomenon can be further exploited to address biological questions relating to the organisms. Here, we aim to provide both biologists and electrochemists with a review of the progress of BPV development with a focus on biological materials, electrode design and interfacial wiring considerations, and propose steps for driving the field forward.

## Graphical abstract

**Forward thinking**: Biophotovoltaic (BPV) systems utilise oxygenic photosynthetic microorganisms associated with an anode to generate an extracellular electrical current, which is stimulated by illumination. The aim of this Minireview is to provide both biologists and electrochemists with an overview of the progress of BPV development with a focus on biological materials, electrode design and interfacial wiring considerations, and propose steps for driving the field forward.

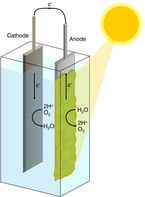

## 1 Introduction

Biophotovoltaic systems (BPVs, also known as photomicrobial fuel cells or microbial solar cells) are devices in which oxygenic photosynthetic micro‐organisms, such as eukaryotic microalgae or cyanobacteria (also known as blue‐green algae), are used to convert sunlight into electricity.^1^ The very first such systems were described at least forty years ago.^2^ Unlike other bioelectrochemical systems such as microbial fuel cells (MFCs) that require an organic substrate to fuel the living organisms, BPVs use the most abundant readily available sources of energy and electrons available on Earth ‐ light^3^ and water. While ‘anoxygenic’ photosynthetic bacteria, such as the purple non‐sulphur bacterium *Rhodopseudomonas*, have been used in MFCs^4^ they are not able to use water as an electron source, and will not be considered here.

Oxygenic photosynthetic micro‐organisms extract electrons from water using light energy, catalysed by photosystem II (PSII).^5^ These electrons are transferred through the photosynthetic electron transfer chain (PETC) to produce NADPH and generate an electrochemical gradient to drive ATP production.^6^ However, some electrons derived from photosynthetic electron transfer pass from the thylakoid to the cytoplasmic or plasma membrane and then outside the cell, in a phenomenon termed ‘exoelectrogenesis’. Other metabolic processes, such as respiration, may also contribute to exoelectrogenesis.^7^

**Figure 1:**
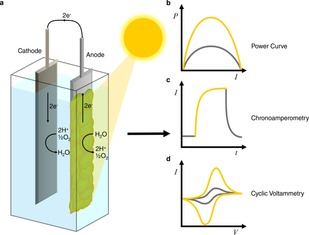
Schematic representation of a biophotovoltaic system (a), a power curve (b), results from chronoamperometry (c), and cyclic voltammetry measurements (d). The gold lines indicate values observed under illumination, and the grey lines indicate values observed in the dark. The cyclic voltammetry measurements correspond to a situation where illumination stimulates release of a redox species. See text for more details.

In a simple two‐electrode biophotovoltaic device (Figure 1a), electrons exported from the micro‐organisms reach an anode via direct and/or mediated indirect electron transfer (defined below) and pass via an external circuit to the cathode. There, they reduce oxygen and protons (which diffuse from the anode and in some instances pass through an ion‐conductive membrane or salt bridge between the anode and cathode) to form water. The process is driven by the potential difference between the anodic and cathodic redox reactions.

Power output from BPV systems is often assessed using a power curve (Figure 1b), showing the external power delivered as a function of current.^8^ The power output can be used directly to report on parameters that affect the physiology of the organisms involved, allowing for the use of BPVs as environmental biosensors.^9^ Alternatively the power can be used to run external electrical devices. The electron transfer kinetics and bioenergetics can be analysed using techniques such as chronoamperometry (Figure 1c) and cyclic voltammetry (Figure 1d).^10^ These techniques involve the measurement of the current generated over time, during light and dark cycles, and during change in applied potential respectively.

We summarise here the state‐of‐the‐art in BPVs, and some of the developments that have led to this point. We will first introduce biological materials commonly used in BPV studies, then the techniques used to assess performance. We then examine electrode materials and architecture used in BPV, and discuss systematically how cells can be ‘wired’ to electrodes. We discuss the limitations of some studies and where future developments may be expected.

## 2 Features of Biophotovoltaic Systems

### 2.1 Biological Materials

Most biophotovoltaic studies use cyanobacteria, either as pure cultures^11^ or environmental samples.^12^, ^13^ Many different cyanobacterial strains have been used, but probably the most frequently used individual one is the model organism *Synechocystis* sp. PCC6803 (hereafter referred to as *Synechocystis*).^10^, ^14^, ^15^, ^16^, ^17^, ^18^, ^19^, ^20^ It is a unicellular coccoid organism, with diameter around 1.6 μm, although this is altered in some mutant strains.^21^ Some cyanobacteria, such as *Synechococcus elongatus* PCC7942,^22^, ^23^ which is also commonly used, have more elongated cells. Others, such as *Nostoc*,^24^, ^25^ routinely form filaments.

Some studies have used eukaryotic algae, predominantly either green algae, notably various species of *Chlamydomonas*,^26^, ^27^, ^28^, ^29^, ^30^ *Chlorella* 31, 32, 33, 34 and *Dunaliella*,^35^ or diatoms, such as *Phaeodactylum tricornutum* and *Thalassiosira pseudonana*.^28^ Green algae and diatoms are only very distantly related in evolutionary terms.^36^ Although cells of the eukaryotic green alga *Ostreococcus tauri* ‐ possibly the smallest eukaryote known ‐ are only around 1 μm in diameter,^37^ eukaryotic algal cells are typically larger than those of cyanobacteria. Cells of the widely used model green alga *Chlamydomonas reinhardtii* are about 10 μm in diameter, although this varies during the cell cycle.^38^

**Figure 2:**
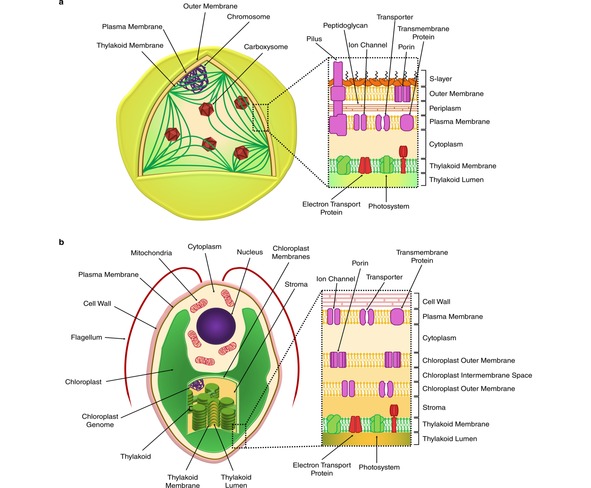
Schematic representation of the model cyanobacterium *Synechocystis* (a) and the eukaryotic microalga *Chlamydomonas* (b), showing the organisation of the photosynthetic thylakoid membranes and external membranes.

In most cyanobacteria, the primary light‐dependent electrogenic reactions occur in thylakoid membranes that, although intracellular, are probably continuous with the cytoplasmic membrane bounding the cell.^6^ The respiratory electron transfer chain is also located in the thylakoid membrane in cyanobacteria, and there may additionally be respiratory electron transfer complexes in the cytoplasmic membrane.^6^ Outside the cytoplasmic membrane is a periplasmic space (also containing many redox active proteins as well as a peptidoglycan layer), an outer membrane, and an additional layer (the S‐layer) composed of protein and polysaccharide (Figure 2a). Cells are sometimes embedded in a matrix of secreted polysaccharide.

Eukaryotic algal cells (Figure 2b) contain a membrane‐bound organelle, the chloroplast (or plastid), which is the site of the photosynthetic thylakoid membranes. The fundamental light‐driven electron transfer reactions in chloroplasts, including the oxidation of water, are very similar to those in cyanobacteria, as chloroplasts ultimately owe their origin to the endosymbiotic acquisition of a cyanobacterium by a non‐photosynthetic cell.^39^ However, the fact that photosynthesis in eukaryotic algae occurs in a discrete, membrane bound, subcellular compartment means there are important differences between cyanobacteria and algae in the topological relationship between the primary electrogenic membranes and an external electrode (Figure 2). In eukaryotic algae, the primary photosynthetic complexes are separated from the rest of the cell by two (for green and red algae, and up to four in some evolutionary lineages) chloroplast envelope membranes, with an additional membrane, the plasma membrane, surrounding the cell. This is further surrounded by a cell wall, whose composition varies widely among species. It comprises polysaccharides, proteins and in some groups, such as the diatoms, complex silicaceous structures.

In addition to intact cells, studies have been carried out with purified photosystems (the protein complexes that perform the photochemistry of photosynthesis),^14^ thylakoid membranes,^15^, ^40^ isolated chloroplasts,^41^ and cyanobacterial cells subjected to mild physical stress.^42^

There is a wide range of growth conditions and media for cyanobacteria and eukaryotic algae. Growth conditions can influence exoelectrogenic activity. For example, iron limitation was reported to lead to an increase in exoelectrogenic activity in *Synechococcus elongatus* PCC7942.^43^ Cells can be grown in suspension (sometimes called planktonic mode) or in layers on a surface such as an electrode. Although these are often referred to as ‘biofilms’, they do not necessarily show the complex structure of many microbial biofilms, such as those of pathogenic *Pseudomonas* strains. Using biofilms may offer more direct and efficient charge transfer between the cells and the electrode, reducing energy loss.

There have been few systematic experiments on the longevity of photosynthetic microorganisms in BPVs, but McCormick *et al*.^35^ reported biofilms of *Chlorella vulgaris* and *Synechococcus sp*. WH5701 producing power throughout a 32‐day experiment, and Bateson *et al*. described a system made with re‐used plastic bottles containing *Chlorella sorokiniana* that remained active over a 35‐day experiment.^44^ By contrast, thylakoid membranes and isolated photosynthetic components only function for minutes to hours depending on conditions, as they do not have the capacity to self‐repair or reproduce.^15^, ^45^

Genetic modification has been used to study exoelectrogenic activity. Although not as electrogenically active as electrogens that are not model strains,^46^ *Synechocystis* can be readily genetically manipulated.^47^ This, and the good understanding of the bioenergetics of this organism, are the main reasons for its widespread use in BPV studies. Other species, such as *Synechococcus elongatus* PCC7942, can also be genetically manipulated. Although tools for genetic manipulation of eukaryotic algae are not as well developed as for those cyanobacteria, they are well developed for *Chlamydomonas reinhardtii* 48 and there are already substantial mutant collections available for some.^30^ Previous genetic engineering strategies include removing known electron sinks such as the respiratory terminal oxidases,^49^ and integrating components of the exoelectrogenic pathways of powerful exoelectrogens such as *Shewanella* sp. and *Geobacter* sp..^22^

### 2.2 Two‐Electrode Systems

**Figure 3:**
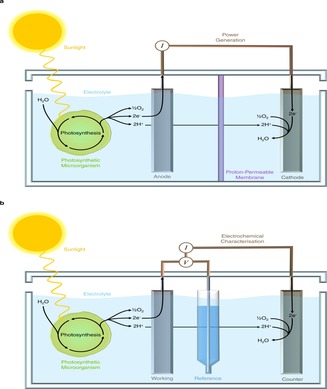
Schematic representation of two‐electrode (a) and three‐electrode (b) biophotovoltaic systems.

The photoelectrochemistry of photosynthetic microorganisms can be studied using either a two or three‐electrode system. As noted previously, a two‐electrode configuration involves the photosynthetic microorganisms making electrical contact with an anode that is connected via an external circuit to a cathode (Figure 3a). The two electrodes can be localised together in one chamber in electrolyte or localised separately in two chambers, which are separated by a proton‐permeable membrane to isolate the cathode from the photosynthetic microorganisms. (Such a membrane is more likely to be used if the microorganisms are in a planktonic state.)

Resistors of varying magnitude and a voltmeter can be connected in parallel in the external circuit and used to determine the current in the entire electrochemical cell at different external resistances. Alternatively, a potentiostat can be used to apply a bias potential between the anode and cathode and similarly measure the current of the connected electrochemical cell. Measurement of the current flowing as a function of the external resistance or voltage applied allows one to determine the power output obtainable, and this is known as a power curve (Figure 1b). The peak power output is a widely used measure of the performance of biophotovoltaic devices.^8^ Note that while measurements made by a two‐electrode configuration provide holistic information about the cell, electrochemical information involving individual electrodes of the biophotovoltaic device may be masked. It may therefore be difficult to identify electron transfer bottlenecks and mechanisms using this regime.

Nevertheless, the two‐electrode configuration has the most straightforward set up, and provides direct information on the total power output for a BPV. It is therefore most suitable for use in studies of factors that affect the overall power output of a device and in field applications. For example, two‐electrode BPVs were used to demonstrate the role of Photosystems I and II in power output^15^ and drive a small digital clock.^35^ Bateson *et al*. constructed a two‐electrode device with an anode made of re‐used aluminium cans in a container made of a re‐used plastic bottle, that harnessed the exoelectrogenic activity of *Chlorella sorokiniana*.^44^ Chouler *et al*. used a two‐electrode device with a mixed culture primarily composed of *Scenedesmus obliquus* and *Chlorella luteoviridis* as a biosensor that showed a decreased power output in response to the addition of formaldehyde.^50^

### 2.3 Three‐Electrode Systems

Three‐electrode systems are commonly employed in the fields of analytical chemistry and protein‐film electrochemistry^51^, ^52^ to provide quantitative information about redox processes at the electrode surface. Although BPV devices may be made up of two‐electrodes, three‐electrode systems are also used by the BPV community to study fundamental questions relating to the photosynthetic microorganisms and the bio‐anode interface. A three‐electrode system allows for the potential applied to one electrode, referred to as the working electrode, to be precisely controlled relative to a reference electrode via a potentiostat.^53^ To study BPV systems, the working electrode is typically the photosynthetic bio‐anode. Calomel or Ag/AgCl electrodes are typically employed as the reference because they have a well‐characterised electrochemical potential relative to that of the standard hydrogen electrode (SHE). An inert counter electrode is employed to discharge currents arising from the working electrode (Figure 3b). For both two‐ and three‐electrode BPV systems, whether one is studying the overall power output or the behaviour of the anode (working electrode), it is important that these parameters are not limited by the cathode (counter electrode). For example, platinum mesh can be used as a counter electrode as it offers a high surface area.

Using the three‐electrode configuration, various informative electrochemical techniques can be applied to analyse the bio‐anode. For example, chronoamperometry is a technique in which the current output of the bio‐anode is measured over time at a constant applied potential, giving information on how the photosynthetic microorganism expels electrons in response to different conditions, such as in different light intensities, in real time (Figure 1c). Cyclic voltammetry is a technique in which the current output is measured over time as the applied potential is cycled, giving information on the redox species in the extracellular space (Figure 1d). Although these experiments can be carried out using a two‐electrode system, the main advantage of using three‐electrode systems to study fundamental questions is that the presence of a reference electrode gives more certainty about the thermodynamics and corresponding kinetics of the redox processes occurring at the working electrode for the duration of an experiment. For complex systems such as biofilms, the outputs generated are then more straightforward to interpret since the variables can be more systematically minimised.

One of the earliest examples of a three‐electrode BPV system being used to probe biological questions is a study by Cereda *et al*.,^10^ in which the chronoamperometry light‐response of planktonic *Synechocystis* was shown to vary systematically with light intensity. Another example is the study by Zhang *et al*.,^14^ where the electrochemical properties of *Synechocystis* biofilms and PSII protein‐films were systematically compared using a range of electrochemical methods. In this study, redox molecules, including O_2_, and a redox species with a mid‐point potential of 0.34 V vs SHE exiting the biofilm following illumination, could be characterised using cyclic voltammetry. Chronoamperometry was used to determine the changes in the magnitude of the photocurrent following different illumination periods, which may provide hints as to the electron transfer mechanism. A later study also investigated the electrochemical properties of a *Synechocystis* biofilm using a three‐electrode configuration, but with cells that had been treated with a ‘gentle’ physical treatment .^42^ Saper *et al*. also identified a redox species with an anodic peak at 0.25 V vs SHE, which could correspond to a redox species participating in the extracellular electron transfer pathway. In a different study, Lu *et al*.^23^ employed a three‐electrode system to control accurately the redox state of the plastoquinone pool within *Synechococcus* sp. PCC7942 via a transmembrane biocompatible electron mediator. As a result, the circadian clock of the cyanobacterial cells could be artificially tuned via electrochemistry.

### 2.4 Developments in Anode Design

In BPVs, the anode (also known as the working electrode in three‐electrode devices) serves as the collector of the reducing equivalents stemming from the photosynthetic microorganisms. The basic requirements of an electrode are that it should exhibit high electrical conductivity and be electrochemically stable within the potential range under study. If cells are to be grown as a biofilm on the electrode, it should also be biocompatible, have a surface suitable for cell adhesion and be relatively optically transparent to allow photosynthetic microorganisms embedded within the electrode to be illuminated. Scaling up of power generation by BPV devices will require electrode materials that are abundant and of low cost. For fundamental bioelectrochemical studies, using electrodes that are easy to make or obtain will increase their widespread use.

Electrode design can be thought of as having progressed through a number of generations over the last 40 years of studies (Table 1). First generation electrodes in BPVs exhibited the basic characteristics of biocompatibility, conductivity and stability but had simple flat geometries. Flat electrodes are generally used in fundamental studies of exoelectrogenic biofilms and protein films; however, in the case of cyanobacteria, photocurrents stemming from biofilms on flat electrodes are often very low and inconsistent. This limits applications in situations where large, reproducible datasets are required. Improving on this, second generation electrodes exhibited rougher surfaces for improved loading and cell adhesion. Recent progress in electrode design has resulted in a third generation of hierarchically‐structured electrodes with very high effective surface areas that enable dense cell loading with improved cell‐electrode interactions, whilst also exhibiting nano‐roughness to promote stable cell adhesion. These third‐generation electrodes were designed according to the dimensions of *Synechocystis*, with pore and channel sizes appropriate for cell penetration and light transmission, whilst also reducing limitations of mass transport and nutrient diffusion.

**Table 1:** A comparison of the different generations of electrodes used in biophotovoltaic systems and SEM images of representative electrode materials at different magnifications (taken with a Tescan Mira 3).

	1st generation electrode (first reported in 1979)	2nd generation electrode (adopted in the 2000s)	3rd generation electrode (adopted 2010 and beyond)
Design	Simple flat substrates	Nano or micron‐roughness	Porous 3D‐structures.
Examples	Platinum; tin oxide	Carbon cloth; carbon nanotubes; carbon paper; graphite; reduced graphene oxide; thin ITO/FTO films on substrates	FTO‐coated ceramic; hierarchically structured inverse opal mesoporous ITO structures
Advantages	Ease of accessibility	Relatively low cost; commercially accessible, electrochemically inert (for carbon‐based electrodes);	High light transmission; hydrophilic; conductive; versatile; nano‐roughness; and easy to tailor
Disadvantages	Non‐optimised design	Opaque; hydrophobic; relatively low electrical conductivity (for carbon‐based electrodes)	Moderate cost; limited electrochemical window
			
Scanning electron microscopy images	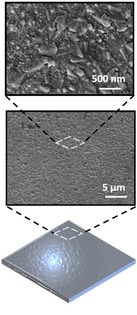	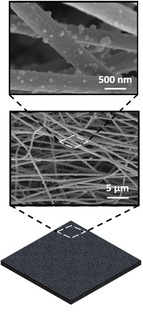	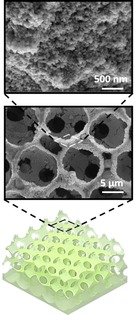
	Platinum	Carbon fibre	IO‐ITO

### 2.5 Electrode Material

Inorganic materials such as stainless steel, copper, platinum and tin oxide were used in electrode fabrication for BPVs as early as the 1970s.^2^, ^20^ Tin oxide is a semiconductor with a conduction band suitable for receiving electrons originating from water oxidation by photosynthesis.^20^ However, carbon‐based materials became more commonly employed in the 2000s because of initial difficulties in introducing nano and micron structures in inorganic materials. Carbon then became the most commonly used anode material in BPVs because of its cheapness, electrical conductivity, robustness, chemical inertness and diversity in form (including nano and micron structures). Different forms of carbon‐based electrodes used in BPVs include carbon cloth,^10^ carbon nanotubes,^24^ graphite^7^ and reduced graphene oxide.^32^ However, carbon has many limitations, and its suitability for use in bioelectrochemical systems has been challenged.^54^ Carbon‐based electrodes are opaque and therefore light penetration to the photosynthetic biofilm may be low. Carbon is also hydrophobic, and may therefore require surface modification to improve bio‐compatibility and wettability. Furthermore, carbon has relatively low electrical conductivity. For example the conductivity of graphite is 3×10^4^ Sm^−1[32]^ and reduced graphene oxide is 15×10^4^ S m^−1^,^55^ whereas copper is 58×10^6^ S m^−1^.^54^, ^56^

In the 2010s, fluorine‐doped tin oxide (FTO) and indium tin oxide (ITO) deposited on glass^15^, ^31^, ^35^, ^57^, ^58^ and ceramic became commonly used, and later ITO nanoparticle‐based electrodes^14^, ^25^ emerged to be the current state‐of‐the‐art anode materials in BPVs. This can be attributed to their optical transparency, nano‐scale surface roughness, hydrophilic surface chemistry, electrical conductivity (1×10^6^ S m^−1^)^59^ and the availability of new fabrication strategies for adapting the structure of the materials into different architectures.^60^ In systematic comparisons of photocurrents produced by cyanobacterial biofilms on different electrodes, ITO electrodes outperformed carbon paper ones in electrical output of biophotovoltaic devices.^31^, ^57^ However, the former are more expensive and indium is a rare‐earth element, making its widespread use undesirable on sustainability grounds.

### 2.6 Anode Architecture

As noted above, the first generation of anodes reported for use with BPVs in the 1980s were simple and planar in design.^2^, ^20^ The second generation comprised thin conductive films that were self‐supported, usually flexible and featured nano‐ or micron‐scale surface roughness to facilitate healthy growth of biofilms. Examples include carbon‐based cloth and paper featuring micron‐sized fibres.^10^, ^57^, ^61^, ^62^ A number of studies have shown that the nanoscale surface morphology of electrodes is essential to create an effective interface with microorganisms.^63^, ^64^

The third generation of electrodes started to emerge in the 2010s, where highly porous (pores were in the millimetre scale) ceramic structures inspired by bone were coated with FTO to offer a high surface area conductive scaffold. This greatly outperformed benchmark carbon‐based electrodes in regard to power density output by biofilms of *Chlorella vulgaris*.^31^ Hierarchical inverse‐opal mesoporous (IO‐meso) ITO electrodes fabricated originally for isolated photosynthetic protein complexes represented another step up.^65^ These hierarchically structured electrodes, which were designed for the physical properties of photosystem II, featured 750 nm macropores and 100 nm interconnecting channels to facilitate the penetration of the biocatalyst, light and electrolyte, and could be fabricated up to thicknesses of up to 80 μm. Importantly, they featured a mesoporous substructure that provided appropriate roughness to aid protein adsorption. This architecture gave an unparalleled 1600‐fold improvement in protein loading and subsequent 3 orders of magnitude improvement in photocurrent compared with flat electrodes. Furthermore, this electrode architecture enabled stable and enhanced integration of a range of redox active guests, including various oxidoreductases and co‐immobilisation with redox polymers.^66^ This hierarchical structure was then adopted for intact *Synechocystis* cells, with 10 μm macropores and 3 μm interconnecting channels, at a thickness of 40 μm.^14^ This gave rise to stable non‐mediated photocurrents (0.3 μA cm^−2^, 680 nm at 1 mW cm^−2^) that grew over 5 days. A study using 40 μm macropores gave similar steady state non‐mediated photocurrents for *Nostoc* (white light, >5 mW cm^−2^).^25^ A key finding in this study was that the nano and meso‐porous sub‐structure, resulting from the use of nanoparticles, was an important factor in the improvement of the photoresponse.

### 2.7 Cell‐Electrode Wiring: Classification of Systems

**Figure 4:**
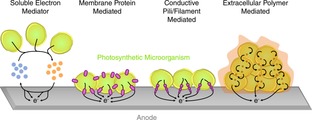
Schematic representation of different mechanisms of cell‐electrode wiring and electron transfer.

In BPVs, ‘wiring’ refers to the electrical connection between the photosynthetic microorganisms and the anode. As with microbial fuels cells, wiring strategies in BPVs can broadly be categorised^1^ into indirect extracellular electron transfer (IEET), where a diffusible mediator transfers electrons from cells to the electrode and direct extracellular electron transfer (DEET), where a non‐diffusible conductive structure such as a membrane‐bound cytochrome, a pilus, or a conductive matrix is responsible for the transfer (Figure 4). For indirect systems, a distinction can be drawn between those where the mediator is generated endogenously within the bioelectrochemical device (but not necessarily by the exoelectrogenic organisms themselves, as with humic substances for example), and those where the mediator is added exogenously.^1^ However, there is increasing use of exogenously applied matrices or substrates for direct electron transfer, as discussed below, so the endogenous/exogenous distinction can be applied to direct mechanisms as well. (As with any nomenclature, some distinctions may be difficult to draw. For example, a system using an endogenous mediator might be supplemented with more of the same mediator added directly and exogenously to it. An alternative distinction from endogenous/exogenous might be whether the mediator was generated naturally or not. However, this distinction is also blurred, as an organic mediator might be synthesised either biologically or chemically.)

We therefore categorise systems as indirect, where a mediator is required, or direct. We also categorise them as endogenous or exogenous. For endogenous systems the electron transfer pathway can in principle be generated without the addition of extra materials directly contributing to the conduction pathway. If extra materials directly contributing to the conduction pathway are required, it would be classified as exogenous. For example, an indirect system using an added redox mediator that was not already being generated by the system would be classified as exogenous. However, a system that generated increased power simply through manipulation of the growth medium, e. g. depletion of a nutrient, would be classified as endogenous (and might be direct or indirect).

### 2.8 Endogenous EET Mechanisms

Understanding of endogenous IEET mechanisms in exoelectrogenic photosynthetic organisms remains limited. McCormick *et al*. considered the best‐described endogenous mediators in other microbial systems, namely phenazines, flavins and quinones.^1^ They noted there was no genomic evidence for synthesis of phenazines by cyanobacteria, or evidence for systems for secretion of quinones or flavins. They did not exclude the possibility of flavins or quinones entering the extracellular medium through cell lysis, but they noted that cyclic voltammetry had not at that time revealed evidence for extracellular redox species. They also suggested a role for reactive oxygen species. Zhang et al^14^ inferred through CV the secretion of a redox species with a midpoint potential of 0.34 V versus SHE, and suggested it may correspond to benzoquinone or flavin derivatives. Saper *et al*. inferred by CV the release of a low molecular weight redox mediator from physically stressed *Synechocystis* and suggested it was a temperature sensitive and water soluble quinone, flavonoid or small peptide.^42^ Certainly in *Shewanella* sp., the secretion of flavin mononucleotide and riboflavin contributes greatly to EET.^67^, ^68^, ^69^ Flavins also mediate EET in hundreds of Gram‐positive bacterial species within the Firmicutes.^70^ A broad range of exogenous quinone analogues can be used to mediate photosynthetic EET^71^ (see below), showing that this class of molecule can in principle function as a mediator. It therefore seems likely that at least some cyanobacteria may use a molecule such as a quinone or a flavin for endogenous IEET. It has also been suggested based on chronoamperometry profiles that transporters or gated ion channels are involved in exporting an endogenous mediator that would otherwise be unable to cross the cytoplasmic membrane.^14^ Identification of any endogenous mediators for IEET is clearly a high priority for the field.

DEET in photosynthetic microorganisms is equally poorly understood. In *Shewanella*, DEET depends on outer‐membrane multi‐haem cytochromes, which may be located in protrusions from the outer membrane.^72^ In *Geobacter* multi‐haem cytochromes are also important, and it has recently been shown that such cytochromes may constitute the extracellular filaments (sometimes referred to as ‘nanowires’) that were previously thought to be conductive pili.^73^ There have been some reports of conductive pili in *Synechocystis*,^74^, ^75^ but the mechanism of conduction is unclear. There is no evidence to date of multihaem *c*‐type cytochromes on the cell surface of cyanobacteria. For photosynthetic microorganisms it will be important to consider also the possible roles of matrix and cell surface structures such exopolysaccharides,^76^ the surface‐layer^77^ and extracellular appendages, such as type IV pili in *Synechocystis* 78 and flagella in eukaryotic microalgae such as *C. reinhardtii*.^79^ However, these may influence factors such as adherence of the cells to the anode, cell‐cell connection in a biofilm, and phototaxis.^79^, ^80^ They may therefore have an effect on DEET without being directly involved in the electron transfer pathway.

### 2.9 Exogenous EET Mechanisms

Planktonic BPV systems usually rely on exogenous mediators for IEET. These are most commonly potassium ferricyanide,^10^, ^15^, ^16^, ^20^, ^26^, ^28^, ^29^, ^31^, ^58^, ^81^ and quinones such as 2,6‐dichloro‐p‐benzoquinone (DCBQ),^14^, ^30^ 2‐hydroxy‐1,4‐naphthoquinone (HNQ)^82^, ^83^, ^84^, ^85^ and 1,4‐benzoquinone^24^, ^86^ (Figure 4). The major limitation of using such exogenous mediators for practical applications is that they may be toxic to the cells and expensive to scale up.^87^ Importantly, they result in the loss of potential during the electron transfer process. For analytical studies, this will also mask electrochemical information.

There is increasing interest in exogenous methods for enhancing DEET. Beyond establishing natural biofilms of photosynthetic microorganisms on the electrode in BPVs, immobilisation of cells in an artificial matrix on the electrode has been proposed as a method for increasing power densities.^33^, ^88^

Artificial matrices can also be designed specifically to be conductive. Particularly impressive results have been obtained by Gorton and co‐workers with polymeric osmium complexes that conduct electrons directly from the cell to the electrode via electron hopping. The polymers also introduce nano‐roughness, helping biofilm formation as well as electron transfer. These systems have been successfully applied to cyanobacteria such as *Leptolyngbia* sp. and eukaryotic green algae such as *Paulschulzia pseudovolvox*, with dramatic enhancements in output.^7^, ^89^, ^90^, ^91^ However, further characterisation of the long‐term effects, if any, of redox polymers on cell physiology will be useful.

## 3 Discussion and Future Directions

### 3.1 Experimental Design and the Difficulty of Comparing Studies

Comparing the results of different studies is difficult, as has recently been noted elsewhere.^92^ However, a comparison is shown in the Supplementary Table. Not all studies reported outputs in IUPAC units for current density (mA m^−2^) and power density, and some studies did not report the electrode surface area so densities could not be calculated to be included in the table. Furthermore, not all studies reported inputs of light intensity and wavelength (nm), temperature (°C), pH and buffering capacity of the electrolyte, and, cell culture conditions and loading. Without these details, it was impossible to compare all previous studies to date. While there are some studies in which BPV components are systematically compared^10^, ^14^, ^31^, ^57^ there is a lack of standardisation between studies. Ideally, all inputs and outputs should be reported (in IUPAC units, and specifying whether outputs are reported as peak or steady‐state values) with standardised biological materials, electrode designs, cell‐electrode wiring and device designs – unless the specific aim of a study is to see the effect of varying one of those BPV components. Identifying the best measure for the amount of biological material is problematic. One possible metric of cell loading is simple biomass. However, and particularly for photosynthesis‐based systems (including whole cells or fractions), chlorophyll content may be more appropriate. It is important to be aware, though, that chlorophyll content per cell may change in response to environmental conditions (or some mutations) and, for organisms with different chlorophyll types (e. g. chlorophylls *a* and *b* in green algae) the ratio between types may also change. Simply reporting the cell loading for the inoculation of the anode in a BPV before a study may also be insufficient. For studies that may involve significant amounts of cell division or death, or using weakly adherent cells, it is advisable to harvest cells from the anode after the study and quantify loading at the end of the experiment.

Although two‐electrode systems form the basis of practical applications such as biosensors, and can be used to generate useful information about the biological systems, it is important to recognise their limitations. Three‐electrode devices allow for the underlying kinetics and thermodynamics of the electron transfer processes at each electrode to be quantitatively studied and controlled, and hence bottlenecks in the overall device to be more easily diagnosed and biological questions to be answered more reliably. Even where a two‐electrode device is the aim, controlled characterisation of the bio‐anode and abiotic cathode using a three‐electrode set‐up before combining them in a two‐electrode device is likely to be helpful.′

Good electrical wiring between the biological material and the anode is also important, or fast kinetic extracellular electron transfer may be missed. Good wiring may be easier to achieve with third‐generation electrodes, revealing complex shapes in the photocurrent profile that may otherwise be difficult to capture.^14^ For all studies, controlled environmental parameters and cell growth conditions are important, and experimental design should be matched with the properties of the system under study. For example, one should avoid employing light intensities that will cause photodamage to the organisms used (unless that is the aim), or overpotentials that may cause unintended electrochemical effects.

### 3.2 Future Improvements for BPV Output

In reviewing the output from biophotovoltaic devices we consider the power output in two‐electrode systems separately from current output in three‐electrode systems. For two‐electrode systems, the highest reported power density outputs come from unconventional microfluidic devices. The highest power density output from a microfluidic biophotovoltaic device was 530 mW m^−2^ by Saar *et al*. in 2018 who used a genetically modified *Synechocystis* strain with fewer internal electron sinks, an Indalloy® anode (made from molten InBiSn alloy) and ferricyanide as mediator.^93^ This was closely followed by Liu and Choi in 2017 who achieved 438 mW m^−2^ with wild type *Synechocystis*, a carbon cloth anode and a PEDOT:PSS redox polymer matrix.^18^ Power density output from a microfluidic biophotovoltaic device without an exogenous mediator added reached 294 mW m^−2^ as described by Bombelli *et al*. in 2015 with wild type *Synechocystis* and an Indalloy® anode.^16^ It should be noted that a small amount of energy is needed to run the flow mechanism, which should be accounted for in the overall device output.

If these microfluidic biophotovoltaic devices are excluded, the highest reported power density outputs for traditional two‐electrode biophotovoltaic systems were achieved by Sekar *et al*. in 2014 using *Nostoc* sp. ATCC 27893 and a carbon nanotubes anode. An output of 100 mW m^−2^ was achieved in the presence of a *p*‐benzoquinone mediator, and 35 mW m^−2^ when no mediator was added.^24^ The highest power density output for a traditional two‐electrode biophotovoltaic system exogenously mediated with a matrix was 6.2 mW m^−2^, which was reported by Luimstra *et al*. (2014) using *Paulschulzia pseudovolvox*, where a carbon‐painted anode and a polypyrrole redox polymer were used.^94^

The highest reported photocurrent density output for a three‐electrode biophotovoltaic system was 600 mA m^−2^ by Longatte *et al*. in 2017 who used a *Chlamydomonas reinhardtii ΔpetA* mutant, a carbon gauze anode and DCBQ as mediator.^30^ The next highest photocurrent density output was 481.5 mA m^−2^, which was achieved by Hasan *et al*. in 2014 using *Leptolyngbya sp. CYN826* and a graphite anode, ferricyanide mediator and an osmium redox polymer matrix.^90^ A three‐electrode system reported by Sekar *et al*. in 2016 gave rise to non‐exogenously mediated photocurrent density of 120 mA m^−2^, using a *Synechococcus* mutant expressing OmcS from *Geobacter sulfurreducens*, and a carbon nanotube anode.^22^

McCormick *et al*. calculated that if BPVs were optimised, then power densities between 0.7 and 7.7 Wm^−2^ (current densities of 0.34 to 2.46 mA cm^−2^) may be achievable,^1^ so there is clearly considerable scope for further improvement.

There are a number of possible areas for improvement of the biological material used. They include the use of strains with genetic alterations (affecting endogenous genes or introducing new ones) to enhance output. This will require improved understanding of the biological basis of exoelectrogenic activity. There has also been little consideration of the possible value of using consortia of strains. For example, combinations of strains that secrete redox mediators with others whose exoelectrogenic output is high in principle but limited by transfer of electrons to the anode may be beneficial. For local implementation of ‘real‐world’ applications, identifying strains that are adapted to the environmental conditions (and therefore probably local themselves) is likely to be important.

Significant improvements may come from enhanced electrode design, and developments from the fields of microbial fuel cells and immobilised photosynthetic systems may be useful to apply to BPVs. Although other materials have been demonstrated to have significant advantages over carbon‐based ones, carbon‐based electrodes should not be neglected in future studies because of their versatility and low cost, and the architecture of carbon anodes could be better designed to enable deeper light penetration. Electrodes with hierarchical nano‐structured architectures have proved to be a promising way forward for increasing the effective surface area and cell loading. It may be beneficial to tailor the electrode architecture to the size and shape of other micro‐organisms as well as *Synechocystis*. Biological structures such as those found in bone and other tissues, as well as photonic structures may provide more ideas for electrode architectures, and as well as light concentration and cell attachment.^95^

However, there is a pressing need to devote more attention to the cathode. This is typically made of platinum or platinised carbon, exploiting platinum's catalytic activity for oxygen reduction. Platinum is expensive, though, even when used as a modification of another substrate such as carbon paper, and its use is not sustainable.^60^ It will therefore be important to consider other possible cathodes. Call *et al*.^96^ showed that a MFC using the anoxygenic photosynthetic bacterium *Rhodopseudomonas palustris* in the anode was able to function with an air cathode of graphene‐coated stainless steel. Although the power output was around a quarter of that with a platinum cathode, it was around 500 times greater than that with a stainless‐steel cathode. This suggests that graphene systems may be feasible as cheap cathodes for BPVs. Identification of cheap and sustainable cathodes with high catalytic activity will certainly be crucial for scale‐up of BPVs. Biocathodes are an exciting possibility for this. Berk and Canfield described a system using the anoxygenic photosynthetic bacterium *Rhodospirillum rubrum* at the anode, and a cyanobacterium from the family Oscillatoriaceae acting as a catalyst at the cathode.^97^ Cai *et al*. described a fuel cell system with the cyanobacterium *Microcystis aeruginosa* IPP at the cathode. The cyanobacterial cells generated reactive oxygen species, which in turn served as electron acceptors from the cathode.^98^

In determining how to scale up biophotovoltaic devices for practical applications, it may be possible to learn lessons from work with MFCs. One approach that may be useful for BPVs is combining multiple cells in a single installation rather than scaling up individual cells. Multiple cells were used in a MFC installation designed for large‐scale processing of urine to provide lighting for the urinals at the Glastonbury Music Festival.^99^ The design required development of an appropriate system for power handling (including responding to differences in output from individual cells, and boosting the voltage to drive the LEDs for illumination), which may also be valuable for BPVs. Similar MFC installations are being tested for sanitary processing in developing countries. MFCs are intrinsically limited by the mass transport of the organic substrates required for feeding the heterotrophic bacteria on which they operate, and work with MFCs has also considered how to improve mass transport. However, this is less relevant to BPVs, which are fed by light and water rather than organic substrates. For BPVs, light penetration is instead more of a concern, and this could be enhanced with an appropriate electrode architecture.

Scaling up of BPVs for real‐world applications will raise broader challenges as well as technical ones. These range from questions of sustainability (the need for life cycle analyses) to implementation research (how to deal with installation and maintenance in a way that is sensitive to the needs of the people and areas involved). For these, a truly interdisciplinary approach will be essential.

## Abbreviations

BPV: biophotovoltaic system
CV: cyclic voltammetry
DEET: direct extracellular electron transfer
EET: extracellular electron transfer
FTO: fluorine‐doped tin oxide
IEET: indirect extracellular electron transfer
IO: inverse opal
ITO: indium tin oxide
MFC: microbial fuel cell
NADPH: nicotinamide adenine dinucleotide phosphate
PETC: photosynthetic electron transfer chain
PQ: plastoquinone
PSII: photosystem II
SHE: standard hydrogen electrode

## Conflict of interest

The authors declare no conflict of interest.

## Biographical Information

*Laura Wey graduated from the Australian National University (Australia) with a Bachelor of Philosophy (PhB) in molecular biology. She then worked at the ARC Centre of Excellence for Translational Photosynthesis (Australia) on cyanobacterial bicarbonate transporter‐based CO_2_ concentrating mechanisms. She is now a PhD student under the supervision of Prof. Christopher Howe in the Department of Biochemistry at the University of Cambridge (UK), researching the biological origins of exoelectrogenesis in cyanobacteria*.

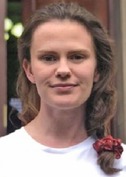

## Biographical Information

*Paolo Bombelli is a postdoctoral researcher in the Department of Biochemistry at the University of Cambridge (UK), where his work on biophotovoltaics has attracted several collaborations from a range of academic partners, including the Zoological Society of London, and commercial interest. He holds an MSc in plant biology from the University of Milano‐Statale (Italy) and a PhD in Chemical Engineering from the University of Cambridge (UK). His talents are multidisciplinary, and he has worked across biophysics, microbiology and agriculture. He currently holds the desirable title of ‘algal electrician’*.

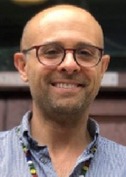

## Biographical Information

*Xiaolong Chen is a postdoctoral researcher at the Department of Chemistry in the University of Cambridge (UK). He is conducting research in tailoring high‐surface‐area electrodes for biocatalysts used in biophotovoltaic and microbial fuel cells. He achieved an MSc in advanced manufacturing from the University of Manchester (UK) and a PhD in electrochemical metal 3D printing from Imperial College London (UK)*.

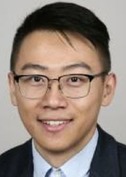

## Biographical Information

*Joshua Lawrence is a PhD student at the University of Cambridge (UK), working under the supervision of Prof. Christopher Howe and Dr. Jenny Zhang. He graduated from Imperial College London (UK) with a BSc in Biotechnology, focusing on synthetic, systems and structural biology. His research concentrates on using a synthetic biology approach to engineer highly exoelectrogenic cyanobacteria*.

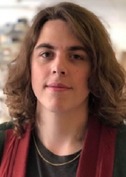

## Biographical Information

*Clayton Rabideau graduated from the University of London, Queen Mary (UK) with a BSc in Genetics. He is currently a PhD student under the supervision of Professor John S. Dennis in the Department of Chemical Engineering and Biotechnology at the University of Cambridge (UK). His primary research interest is the development of systems for using exoelectrogenesis to probe metabolism, remediate waste, and generate electrical power*.

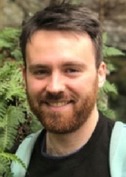

## Biographical Information

*Stephen Rowden is a postdoctoral researcher in the Biochemistry Department of the University of Cambridge (UK), based in the lab of Professor Christopher Howe. His current work focuses primarily on the use of electrochemistry to manipulate/control aspects of cellular physiology in cyanobacteria. He worked previously as a postdoctoral researcher at the University of Greenwich, London (UK) with Professor Pat Harvey on a sustainable CO_2_ algae biorefinery. He holds a BSc (Hons) in Molecular Biology from Queen Mary, University of London (UK), an MRes in Biochemical Research from Imperial College London (UK) and a PhD in Biochemistry from the University of Cambridge*.

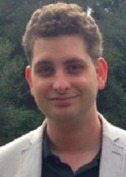

## Biographical Information

*Jenny Zhang graduated from The University of Sydney (Australia) with a PhD in bioinorganic chemistry. She then joined Professor Erwin Reisner as a Marie Sklodowska‐Curie postdoctoral fellow to work on semi‐artificial photosynthesis at The University of Cambridge (UK). She is now a David Phillips Fellow and group leader at the same institution, developing biophotoelectrochemical platforms for the study of photosynthetic microorganisms*.

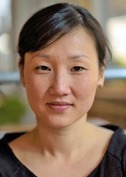

## Biographical Information

*Christopher Howe is Professor of Plant and Microbial Biochemistry in the Department of Biochemistry at the University of Cambridge (UK), and holds a PhD and ScD from the University of Cambridge (UK). His research interests cover a wide range of topics in photosynthesis, ranging from the basic biochemistry of photosynthetic electron transfer to the evolution of chloroplast genomes, and including the biotechnological exploitation of photosynthetic organisms*.

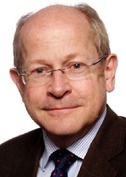

## Supporting information

As a service to our authors and readers, this journal provides supporting information supplied by the authors. Such materials are peer reviewed and may be re‐organized for online delivery, but are not copy‐edited or typeset. Technical support issues arising from supporting information (other than missing files) should be addressed to the authors.

Supplementary
