# Supplementary material for: The Development of Biophotovoltaic Systems for Power Generation and Biological Analysis
Source: ChemElectroChem. 2019 Sep 18;6(21):5375–86. doi: 10.1002/celc.201900997 (PMC6899825; doi:10.1002/celc.201900997)
Supplement: Supplementary file 1 — Supplementary [file CELC-6-5375-s001.pdf]

CHEMELECTROCHEM

Supporting Information

© Copyright Wiley-VCH Verlag GmbH & Co. KGaA, 69451 Weinheim, 2019

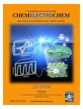

The Development of Biophotovoltaic Systems for Power Generation and Biological Analysis

Laura T. Wey, Paolo Bombelli, Xiaolong Chen, Joshua M. Lawrence, Clayton M. Rabideau, Stephen J. L. Rowden, Jenny Z. Zhang,* and Christopher J. Howe*© 2019 The Authors. Published by Wiley-VCH Verlag GmbH & Co. KGaA.
This is an open access article under the terms of the Creative Commons Attribution License, which permits use, distribution and reproduction in any medium, provided the original work is properly cited.An invited contribution to a Special Collection dedicated to Bioelectrochemistry.

Supporting information

Supplementary Table 1. A comparison of power density outputs for two-electrode biophotovoltaic systems in different studies. (MUT) denotes a mutant strain was used.

Photosynthetic microorganism	Exogenous mediator	Anode Material	Anode Structure	Anode generation	Maximum Power output (mW m^{-2})	Year	Ref.
Filamentous cyanobacteria	NA	Platinum	Flat	1st	0.132	2009	100
Filamentous cyanobacteria	NA	Platinum	Flat	1st	5	2010	101
Synechocystis	5 mM Ferricyanide	ITO-coated PET	Flat	2nd	0.696	2011	15
Synechocystis	NA	ITO-coated PET	Flat	2nd	0.114	2011	35
Chlorella	NA	ITO-coated PET	Flat	2nd	0.45	2011	35
Dunaliella	NA	ITO-coated PET	Flat	2nd	7	2011	35
Synechococcus	NA	ITO-coated PET	Flat	2nd	10	2011	35
Chlorella	2.5 mM Ferricyanide	Carbon Cloth	Fibrous	2nd	0.2	2011	31
Chlorella	2.5 mM Ferricyanide	FTO-coated ceramic	Hierarchically Structured Porous	3rd	14	2011	31
Chlorella	2.5 mM Ferricyanide	FTO-coated glass	Flat	2nd	24	2011	31
Filamentous cyanobacteria	NA	Carbon Paper	Flat	2nd	2.05	2012	57
Filamentous cyanobacteria	Polyaniline Redox Polymer	Glass	Flat	1st	2.7	2012	57
Filamentous cyanobacteria	NA	Stainless Steel	Flat	1st	5.05	2012	57
Filamentous cyanobacteria	NA	ITO-coated PET	Flat	2nd	23.6	2012	57
Synechocystis	1 mM Ferricyanide	ITO-coated PET	Flat	2nd	0.04	2012	58
Synechocystis (MUT)	1 mM Ferricyanide	ITO-coated PET	Flat	2nd	0.2	2012	58

Photosynthetic microorganism	Exogenous mediator	Anode Material	Anode Structure	Anode generation	Maximum Power output (mW m^{-2})	Year	Ref.
Synechocystis	NA	Carbon Fibre	Flat	2nd	0.01	2012	17
Chlamydomonas (MUT)	NA	Graphite	Flat	2nd	0.82	2012	102
Chlamydomonas	NA	Platinum-coated PET	Flat	2nd	0.15	2013	27
Filamentous cyanobacteria	NA	Gold	Porous	2nd	10	2013	103
Chlamydomonas	NA	Graphite	Flat	2nd	4.741	2013	29
Chlamydomonas	NA	Graphite	Flat	2nd	12.947	2013	29
Filamentous cyanobacteria	NA	ITO-coated Glass	Flat	2nd	0.0248	2013	104
Chlorella	NA	ITO-coated Glass	Flat	2nd	0.112	2014	105
Filamentous cyanobacteria	NA	ITO-coated Glass	Flat	2nd	0.121	2014	105
Chlorella	NA	ITO-coated Glass	Flat	2nd	0.124	2014	105
Synechococcus	NA	ITO-coated Glass	Flat	2nd	0.313	2014	105
Chlorella	NA	ITO-coated Glass	Flat	2nd	0.13	2014	32
Chlorella	NA	Reduced Graphene Oxide-coated Glass	Porous	2nd	0.27	2014	32
Filamentous cyanobacteria	NA	Carbon Nanotubes on Carbon Paper	Nanotubes	2nd	35	2014	24
Filamentous cyanobacteria	2 mM *p*-Benzoquinone	Carbon Nanotubes on Carbon Paper	Nanotubes	2nd	100	2014	24
Synechocystis	NA	Gold-deposited PMMA	Flat	1st	0.0709	2014	19

Photosynthetic microorganism	Exogenous mediator	Anode Material	Anode Structure	Anode generation	Maximum Power output (mW m^{-2})	Year	Ref.
Volvox	Polypyrrole Redox Polymer	Carbon Paint	Flat	2nd	6.2	2014	94
Synechococcus	NA	Gold	Flat	1st	0.0057	2014	106
Synechococcus	NA	Gold	Flat	1st	0.0058	2014	106
Synechocystis	NA	Indalloy®	Flat	2nd	294	2015	16
Chlamydomonas	NA	Graphite	Flat	2nd	0.0015	2015	26
Chlamydomonas (MUT)	NA	Graphite	Flat	2nd	0.0084	2015	26
Synechocystis	NA	Carbon-deposited PMMA	Flat	2nd	0.9	2015	107
Synechocystis	NA	Carbon Cloth	Fibrous	2nd	8	2016	108
Synechocystis	NA	Carbon Nanotubes	Nanotubes	2nd	0.105	2017	109
Synechocystis	NA	Carbon Nanotubes	Nanotubes	2nd	0.38	2017	109
Synechocystis	NA	Carbon Cloth	Flat	2nd	16	2017	18
Synechocystis	NA	Carbon Cloth and Graphite-PTFE Composite	Flat	2nd	175	2017	18
Synechocystis	PEDOT:PSS Redox Polymer	Carbon Cloth	Flat	2nd	438	2017	18
Synechocystis	NA	Carbon Cloth-coated PTFE	Fibrous	2nd	27	2017	110
Filamentous cyanobacteria	NA	Carbon Cloth-coated PTFE	Fibrous	2nd	60.05	2017	1010
Synechocystis	30 mM Ferricyanide	Indalloy®	Flat	2nd	260	2018	93
Synechocystis (MUT)	30 mM Ferricyanide	Indalloy®	Flat	2nd	530	2018	93

Supplementary Table 2. A comparison of photocurrent density outputs for three-electrode biophotovoltaic systems in different studies. (MUT) denotes a mutant strain was used.

Photosynthetic microorganism	Exogenous mediator	Anode Material	Anode Structure	Anode generation	Applied Bias Potential (V vs SHE)	Photocurrent Magnitude (mA m^{-2})	Year	Ref.
Synechocystis	1 mM Ferricyanide	ITO on Plastic PET	Flat	2nd	850	0.0694	1983	20
Synechocystis	NA	Carbon Cloth	Fibrous	2nd	237	4	2014	10
Synechocystis	5 mM Ferricyanide	Carbon Cloth	Fibrous	2nd	497	4	2014	10
Filamentous cyanobacteria	NA	Graphite	Flat	2nd	550	13	2014	90
Filamentous cyanobacteria	1 mM Ferricyanide	Graphite	Flat	2nd	550	59.2	2014	90
Filamentous cyanobacteria	Osmium Redox Polymer	Graphite	Flat	2nd	550	86.4	2014	90
Filamentous cyanobacteria	1 mM Ferricyanide & Osmium Redox Polymer	Graphite	Flat	2nd	550	481.5	2014	90
Volvox	NA	Graphite	Flat	2nd	550	0.2	2015	91
Volvox	Osmium Redox Polymer	Graphite	Flat	2nd	550	3.6	2015	91
Volvox	0.5 mM Ferricyanide & 0.5 mM *p*-Benzoquinone & Osmium Redox Polymer	Graphite	Flat	2nd	550	7.9	2015	91
Volvox	0.5 mM Ferricyanide	Graphite	Flat	2nd	550	18.2	2015	91
Volvox	0.5 mM Ferricyanide & 0.5 mM *p*-Benzoquinone	Graphite	Flat	2nd	550	50.2	2015	91
Volvox	0.5 mM *p*-Benzoquinone	Graphite	Flat	2nd	550	69.7	2015	91
Synechococcus	NA	Multi-walled Carbon Nanotubes on Carbon paper	Nanotubes	2nd	300	10	2016	22

Photosynthetic microorganism	Exogenous mediator	Anode Material	Anode Structure	Anode generation	Applied Bias Potential (V vs SHE)	Photocurrent Magnitude (mA m^{-2})	Year	Ref.
Synechococcus (MUT)	NA	Multi-walled Carbon Nanotubes on Carbon paper	Nanotubes	2nd	300	120	2016	22
Synechococcus (MUT)	0.001 mM *p*-Benzoquinone	Multi-walled Carbon Nanotubes on Carbon paper	Nanotubes	2nd	300	470	2016	22
Filamentous cyanobacteria	Osmium Redox Polymer	Graphite	Flat	2nd	600	10	2017	7
Filamentous cyanobacteria	0.5 mM p-Benzoquinone	Graphite	Flat	2nd	600	320	2017	7
Filamentous cyanobacteria	0.5 mM p-Benzoquinone & Osmium Redox Polymer	Graphite	Flat	2nd	600	480	2017	7
Chlamydomonas	0.1 mM 2,6-DCBQ	Carbon Gauze	Porous	2nd	850	600	2017	30
Synechocystis	NA	ITO	Hierarchically Structured Inverse-Opal	3rd	300	2.1	2018	14
Synechocystis	1 mM DCBQ	ITO	Hierarchically Structured Inverse-Opal	3rd	500	147	2018	14
Synechocystis	NA	ITO on FTO-coated Glass	Flat	2nd	400	0.04	2018	25
Synechocystis	NA	ITO on FTO-coated Glass	Porous	2nd	400	8.4	2018	25
Filamentous cyanobacteria	NA	ITO on FTO-coated Glass	Hierarchically Structured Inverse-Opal	3rd	400	11.2	2018	25
Synechocystis	NA	ITO on FTO-coated Glass	Hierarchically Structured Inverse-Opal	3rd	400	11.5	2018	25
